# Transcriptome profiling of avian pathogenic *Escherichia coli* and the mouse microvascular endothelial cell line bEnd.3 during interaction

**DOI:** 10.7717/peerj.9172

**Published:** 2020-05-21

**Authors:** Peili Wang, Xia Meng, Jianji Li, Yanfei Chen, Dong Zhang, Haoran Zhong, Pengpeng Xia, Luying Cui, Guoqiang Zhu, Heng Wang

**Affiliations:** 1 College of Veterinary Medicine, Yangzhou University, Yangzhou, Jiangsu, China; 2 Jiangsu Co-innovation Center for Prevention and Control of Important Animal Infectious Diseases and Zoonoses, Yangzhou, Jiangsu, China

**Keywords:** Dual RNA-seq, Host–pathogen interactions, Meningitis, APEC, bEnd.3 cells

## Abstract

**Background:**

Avian pathogenic *Escherichia coli* (APEC), an important extraintestinal pathogenic *E. coli*, causes colibacillosis, an acute and mostly systemic disease involving multiple organ lesions such as meningitis. Meningitis-causing APEC can invade the host central nervous system by crossing the blood–brain barrier (BBB), which is a critical step in the development of meningitis. However, the bacteria-host interaction mechanism in this process remains unclear.

**Methods:**

In this study, we examined *E. coli* and bEnd.3 cells transcriptomes during infection and mock infection to investigate the global transcriptional changes in both organisms using RNA sequencing approach.

**Results:**

When APEC infected the bEnd.3 cells, several significant changes in the expression of genes related to cell junctional complexes, extracellular matrix degradation, actin cytoskeleton rearrangement, immune activation and the inflammatory response in bEnd.3 cells were observed as compared to the mock infection group. Thus, the immune activation of bEnd.3 cells indicated that APEC infection activated host defenses. Furthermore, APEC may exploit cell junction degradation to invade the BBB. In addition, amino acid metabolism and energy metabolism related genes were downregulated and the protein export pathway related genes were upregulated in APEC cultured with bEnd.3 cells, compared to that in control. Thus, APEC may encounter starvation and express virulence factors during incubation with bEnd.3 cells.

**Conclusion:**

This study provides a comprehensive overview of transcriptomic changes that occur during APEC infection of bEnd.3 cells, and offers insights into the bacterial invasion strategies and the subsequent host defense mechanism.

## Introduction

Avian pathogenic *Escherichia coli* (APEC), an important extraintestinal pathogenic *E. coli* (ExPEC), causes colibacillosis, an acute systemic disease that involves multiple organ lesions including respiratory, digestive, vascular and nervous system diseases (Dziva & Stevens, 2008; Ewers et al., 2004). Previous studies have demonstrated that APEC strains with different serotypes (O18, O2, O1) can induce meningitis in newborn mammals, such as mice, with varying degrees of septicemia via pathogenic mechanisms that are similar to those of neonatal meningitis-causing *E. coli* (NMEC) strains (Krishnan et al., 2015; Mellata, Johnson & Curtiss, 2018; Zhu Ge et al., 2014). The APEC XM strain (O2:K1), isolated from the brain of a duck with septicemia and meningitis, was shown to be involved in the systemic infection of 7-day-old ducks and 5-week-old Institute of Cancer Research mice, causing severe meningitis in a neonatal mouse model (Hejair et al., 2017; Ma et al., 2014).

Various bacterial factors have been recognized as potent or putative virulence factors of APEC strains, including adhesins (Fim, Pap and Mat), iron acquisition systems (siderophores), two-component regulatory systems (RstAB system, ArcA/B system), vacuolating autotransporter toxin located in the chromosome, and the ColV plasmid encoded virulence genes (Breland, Eberly & Hadjifrangiskou, 2017; Gao et al., 2012; Johnson et al., 2006; Zhao et al., 2015). In particular, it has been reported that the virulence genes *ibeA* and *gimB*, which contribute to the invasion of host cells, are shared between APEC and NMEC (Barbieri et al., 2013; Peigne et al., 2009). Moreover, the host cell cytosolic phospholipase A_2_ and *E. coli ibeA* gene have been proved to be involved in the invasion of brain microvascular endothelial cells (BMECs) (Das et al., 2001; Maruvada & Kim, 2012; Zhu, Pearce & Kim, 2010). Since APEC is a potential reservoir of ExPEC virulence genes and pathogenic to humans (Rodriguez-Siek et al., 2005), APEC infection may pose a potential risk for zoonotic transfer (Mitchell et al., 2015). *E. coli* usually causes meningitis via several steps involving bacteria–host interactions: entry into the gastrointestinal tract mucosa (Birchenough et al., 2017), invasion of the intravascular space, survival and multiplication in the serum to a particular order of magnitude (Sullivan, Lascolea & Neter, 1982), traversing through the blood–brain barrier (BBB), and ultimately contributing to central nervous system (CNS) complications and neuronal injury (Kim, 2003b; Witcomb et al., 2015). A critical step in meningitic process is the bacterial crossing of the BBB, a structural and functional barrier formed by BMECs, astrocytes and pericytes that blocks the transport of harmful substances and pathogenic microorganisms. Bacteria invade the BBB via intercellular and paracellular pathways as well as Trojan horse mechanisms (Kim, 2003a, 2008). However, the mechanisms involved in the bacteria-host interaction during this process remain unclear.

Bacteria can rapidly reprogram their gene expression networks in response to their constantly changing living environment. During in vivo infection, the bacteria compete with the host for survival or nutrition and gene expression changes are observed in both, which differ from those observed in artificial culture conditions. Dual RNA sequencing (RNA-seq) was first used to simultaneously profile host and pathogen transcriptomes in 2012 in order to better understand the host–pathogen interactions (Westermann, Gorski & Vogel, 2012). Since then, dual RNA-seq analyses have been successfully performed to assess pathogen–host interactions, including those between *Pseudomonas plecoglossicida* and *Epinephelus coioides* (Zhang et al., 2019) as well as *Salmonella* and HeLa cells (Westermann et al., 2016). However, the interaction between *E. coli* and BBB-related cells has not yet been explored by dual RNA-seq.

In this study, we investigated the potential mechanism of APEC-host cell interaction by infecting the mouse brain microvascular endothelial cell line (bEnd.3) with the APEC XM strain (O2:K1). The transcriptomes of APEC strain and bEnd.3 cells were measured by dual RNA-seq during the interaction. The findings of this study may contribute toward improving the current understanding of *E. coli* invasion across the BBB.

## Materials and Methods

### Culture conditions

The APEC XM strain (O2:K1) was isolated from the brain of a duck with symptoms of septicemia and meningitis (donated by Dr. Guoqiang Zhu, Yangzhou University), and grown aerobically on Luria-Bertani (LB) plates or in LB broth with agitation (180 rpm/min) at 37 °C. Mouse BMECs (bEnd.3; ATCC CRL-2299, American Type Culture Collection, Manassas, VA, USA) were cultured in Dulbecco’s Modified Eagle Medium (DMEM; Invitrogen, Carlsbad, CA, USA), supplemented with 10% heat-inactivated fetal bovine serum (FBS; Gibco, Carlsbad, CA, USA) in 10 cm cell culture dishes at 37 °C in a 5% CO_2_ atmosphere.

### Bacterial adherence and invasion of bEnd.3 cells

For the adherence and invasion assays, the APEC strain was grown in LB broth with agitation (180 rpm/min) until the optical density at 600 nm reached 1.0 (1 × 10^8^ CFU/mL) in exponential phase. The bacteria were collected by centrifugation (3,500 rpm, 8 min), washed twice with phosphate-buffered saline (PBS), and resuspended in FBS-free DMEM. Then, bEnd.3 cells were infected with the APEC XM strain in 10 cm dishes at a multiplicity of infection (MOI) of 100 for 1, 2, 3, 4, 5 or 6 h at 37 °C in 5% CO_2_. The mock-infection cells were cultured in FBS-free DMEM as control. The bEnd.3 cells were gently washed with PBS three times to remove any non-adherent bacteria, and then lysed with 0.5% Triton X-100 for 30 min at 37 °C. The suspensions were collected, serially diluted 10-fold, and plated on LB plates. After incubation overnight at 37 °C, the number of CFUs was calculated. The time point at which the highest number of bacteria adhered to and invaded the bEnd.3 cells was selected for the sample collection and RNA-seq analysis.

### Total RNA isolation

Bacteria were cultured in DMEM without FBS for 3 h and then were treated with RNAprotect Bacteria Reagent (QIAGEN, Hilden, Germany) to protect the RNA. Total RNA was extracted using TRIzol reagent according to the manufacturer’s instructions (Invitrogen Co., Ltd., San Diego, CA, USA) and genomic DNA was digested using RNase-free DNase. To sequence the bacterial transcriptome, rRNA was removed from the total RNA using a Ribo-Zero rRNA removal kit (gram-negative bacteria, Epicentre Biotechnologies, Madison, WI, USA). The total RNA of bEnd.3 cells, infected with or without APEC for 3 h, were isolated using pre-cooled TRIzol reagent (Invitrogen Co., Ltd., San Diego, CA, USA) according to the manufacturer’s instructions. RNA integrity was analyzed using an Agilent Bioanalyzer 2100 (Agilent Technologies, Palo Alto, CA, USA), RNA purity was checked using a NanoPhotometer^®^ spectrophotometer (IMPLEN, Westlake Village, CA, USA), and RNA concentration was measured using a Qubit^®^ RNA Assay Kit with a Qubit^®^ 2.0 Fluorometer (Invitrogen Co., Ltd., San Diego, CA, USA).

### cDNA library construction and RNA-seq

Nine individual cDNA sequencing libraries (three mock infection APEC samples, three mock-infected bEnd.3 samples, and three infection samples) were prepared using a NEBNext^®^ UltraTM RNA Library Prep Kit for Illumina^®^ (NEB, Ipswich, MA, USA) according to the manufacturer’s recommendations. Index codes were added to attribute sequences to each sample. Briefly, mRNA was purified from total RNA using poly-T oligo-attached magnetic beads and fragmented using divalent cations under high temperatures in NEBNext First Strand Synthesis Reaction Buffer (5×) (NEB, Ipswich, MA, USA). cDNA was synthesized using a random hexamer primer and fragments of 250–300 bp in length were preferentially selected and purified using an AMPure XP system (Beckman Coulter, Brea, CA, USA). PCR was then performed using a Phusion High-Fidelity DNA polymerase (Vazyme, Nanjing, China), universal PCR primers, and Index (X) primers. The PCR products were purified using an AMPure XP system (Beckman Coulter, Brea, CA, USA) and library quality was assessed using an Agilent Bioanalyzer 2100 system (Agilent Technologies, Palo Alto, CA, USA). The index-coded samples were clustered using a cBot Cluster Generation System (Illumina, Inc., San Diego, CA, USA) with a TruSeqPE Cluster Kit v3-cBot-HS (Illumina, Inc., San Diego, CA, USA) according to the manufacturer’s instructions. The library was then sequenced on an Illumina HiSeq platform (Illumina, Inc., San Diego, CA, USA) to generate 125/150 bp paired-end reads. The raw reads in FASTQ format were first processed using in-house Perl scripts and clean data were obtained by removing reads containing adaptor or poly-N sequences and low-quality reads. The Q20, Q30 and GC content of the clean data were then calculated. Bowtie2-2.2.3 was used to build the reference genome index and align the clean reads to the reference genome. HTSeq v0.6.1 was used to count the number of reads mapped to each gene. Gene transcription levels were estimated by calculating the fragments per kilobase of transcript per million mapped reads.

### Differentially expressed genes screening and functional analysis

The DEGs in bEnd.3 cells and APEC were evaluated by comparing the transcriptome data of both cell types cultured in DMEM or during their interaction using the DESeq2 R package (1.16.1). The *P* values of results were adjusted using Benjamini and Hochberg’s approach to control the false discovery rate. Differential expression was determined using the following thresholds: |log_2_-fold change| of ≥1 or 0.5 and an adjusted *P* value of ≤0.05.

DEG functional annotation and enrichment were performed using the Gene Ontology (GO) and Kyoto Encyclopedia of Genes and Genomes (KEGG) databases, while KOBAS software was used to test the statistical enrichment of DEGs in KEGG pathways and the GOseq R package was used to analyze the GO enrichment of DEGs. GO terms with corrected *P* values of less than 0.05 were considered to be significantly enriched for DEGs.

### Quantitative real-time PCR

qRT-PCR was carried out using a previously described method for validating gene expression data obtained by high-throughput profiling platforms (Everaert et al., 2017). A total of 17 genes were randomly selected to analyze their relative expression level and then qRT-PCR primers were designed and validated for these genes (Table S1). qRT-PCR was carried out on a CFX CONNECT Real-time PCR machine (Bio-Rad, Louisville, KY, USA) using ChamQ SYBR qRT-PCR Master Mix (2×) (Vazyme, Nanjing, China) according to the manufacturer’s instructions. The amplification cycles were performed as follows: 95 °C for 10 min, followed by 40 cycles of 95 °C for 30 s, 60 °C for 30 s, and 72 °C for 30 s. All qRT-PCR assays were performed in triplicate, with expression values estimated using the 2^−ΔΔCt^ method and normalized using *gapA* and *GADPH* for APEC and bEnd.3 cells, respectively. The correlation between the fold changes obtained by qRT-PCR and RNA-seq were determined using Pearson correlation analysis.

## Results

### Ability of APEC to adhere to and invade bEnd.3 cells

To characterize the interactions between the APEC strain and the BBB, bEnd.3 cells were used to establish an in vitro model. The ability of adhesion and invasion at six serial time points were evaluated. MOI of 100 was identified as the appropriate infectious dose of bacteria and the maximum adhesion and invasion was achieved at 3 h (Fig. S1).

### Dual RNA-seq analysis of APEC and infected bEnd.3 cells

To characterize the response of bEnd.3 cells to infection and investigate the effect of bEnd.3 cells resistance on the transcriptional response of *E. coli* in vitro, we used a dual RNA-seq method that enabled the simultaneous transcriptional profiling of bacteria and bEnd.3 cells. Total RNA, including cellular and bacterial RNA, was isolated from infected bEnd.3 cells at 3 h. Nine individual cDNA sequencing libraries (three mock infection APEC samples, three mock-infected bEnd.3 samples, and three infection samples) were prepared and sequenced using the Illumina paired-end method, generating more than 1 × 10^7^ clean reads per group after removal of the low-quality reads (Table 1). The clean reads of Q20 and Q30 were above 97% and 91%, respectively, with a GC content of approximately 50%. Next, we mapped the sequenced clean reads to the *Mus musculus* (Ensembl release-92) and *E. coli* (GCF_002844685.1) genomes. In the bEnd.3 cell-APEC interaction (be_AP) group, over 65% of the clean reads were mapped to the *M. musculus* genome and less than 20% were mapped to the *E. coli* genome, providing sufficient data for further analysis. In the two control groups, more than 92% and 99% of the clean reads were mapped to the *E. coli* and *M. musculus* genomes, respectively. All further analyses were based on the uniquely mapped reads and all raw data were submitted to the ZENODO database (DOI 10.5281/zenodo.3689240, 10.5281/zenodo.3672826).

**Table 1:** Summary of illumina RNA-seq data. Each row of data indicates the total reads, clean reads, total mapped clean data, Q30 (%), GC (%), and percent sequence reads mapped of every sample.

Sample	Total reads	Clean reads	Total mapped clean data* (Gb)	Q30 (%)	GC (%)	Mapped rate (%)
APEC_1	11,377,278	11,303,464	1.7G	92.84	51.33	99.43^a^
APEC_2	12,595,278	12,482,110	1.87G	92.86	51.46	99.39^a^
APEC_3	9,850,068	9,786,504	1.47G	94.91	51.45	99.72^a^
bEnd3_1	59,805,426	59,042,776	8.86G	92.05	50.7	92.44^b^
bEnd3_2	49,924,482	49,319,300	7.4G	92.1	51.22	92.46^b^
bEnd3_3	64,453,186	63,528,514	9.53G	91.87	50.15	92.29^b^
bE_AP_1	77,516,134	76,516,858	11.48G	92.15	48.91	19.16^a^
	79,574,798	78570978	11.79G	92.14	49.1	67.44^b^
bE_AP_2	77,558,096	76,635,112	11.5G	92.83	48.42	16.29^a^
	80,037,182	79,108,450	11.87G	92.81	48.63	69.38^b^
bE_AP_3	75,686,054	74,877,152	11.23G	92.91	48.65	13.96^a^
	78,470,990	77,655,916	11.65G	92.89	48.85	71.53^b^

**Notes:**

* Clean data were obtained from raw data by removing reads containing adapter, ploy-N and low quality reads.

a Clean reads were mapped to *Escherichia coli* genome.

b Clean reads were mapped to *Mus musculus* genome.

### Validation of dual RNA-seq data by qRT-PCR

**Figure 1:**
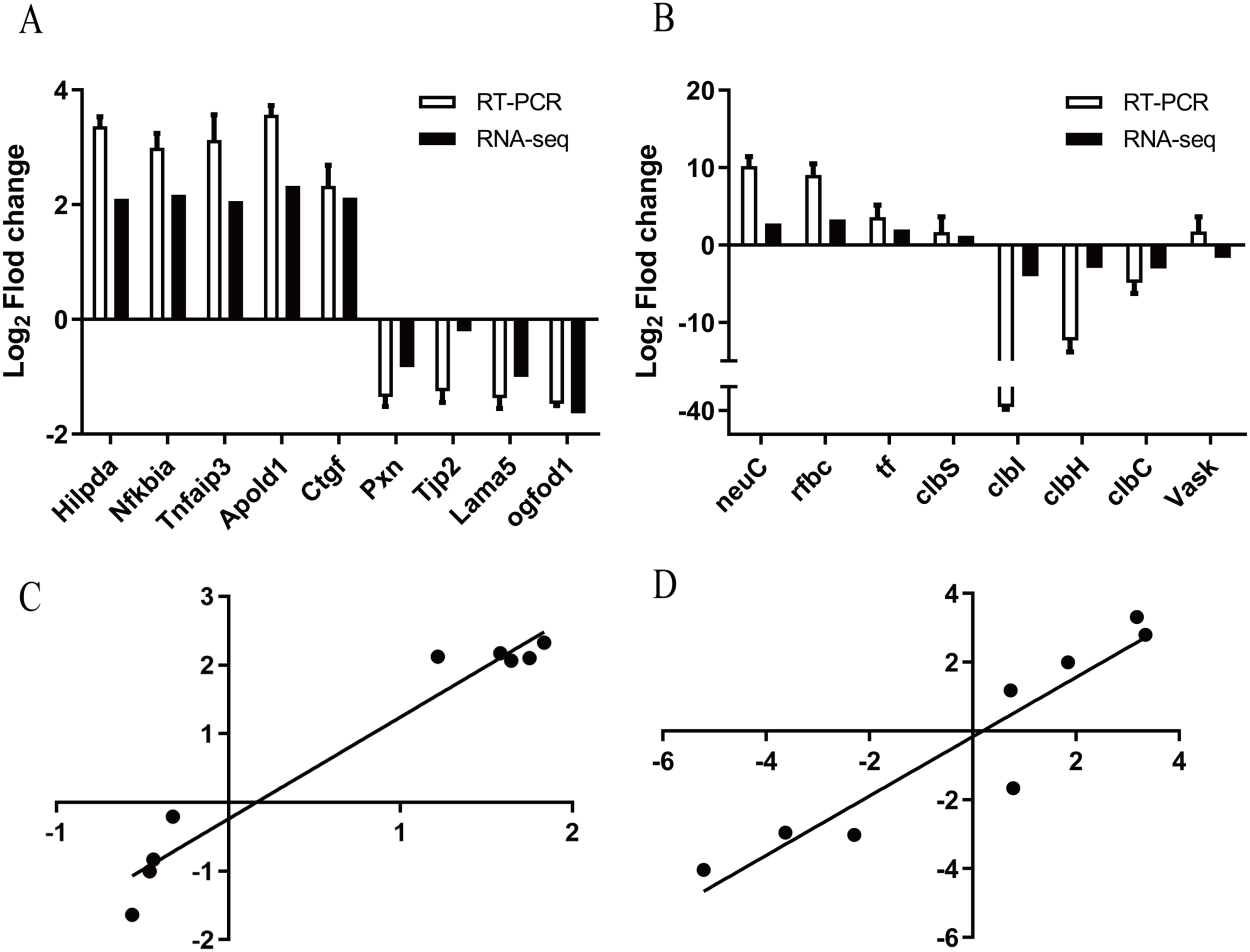
Validation of dual RNA-seq analysis. (A) qRT-PCR analysis of bEnd.3 transcriptome representative genes identified by RNA-seq. The *x*-axis represents individual genes and the *y*-axis fold change in expression determined by RNA-seq (black bars) or qRT-PCR (white bars). All data are shown as means ± SD. BEnd.3 transcriptome representative genes are *Hilpda* (hypoxia inducible lipid droplet associated), *Nfkbia* (nuclear factor of kappa light polypeptide gene enhancer in B cells inhibitor, alpha), *Tnfaip3* (tumor necrosis factor, alpha-induced protein 3), *Apold1* (apolipoprotein L domain containing 1), *Ctgf* (connective tissue growth factor), Pxn (paxillin), *Tjp2* (tight junction protein 2), *Lama5* (laminin, alpha 5) and *Ogfod1* (2-oxoglutarate and iron-dependent oxygenase domain containing 1). (B) qRT-PCR analysis of BEnd.3 transcriptome representative genes identified by RNA-seq. APEC strain transcriptome representative genes are *clbS* (colibactin self-protection protein clbS), *neuC* (UDP-N-acetylglucosamine 2-epimerase (hydrolyzing)), *tf* (type 1 fimbrial protein), *rfbC* (dTDP-4-dehydrorhamnose 3,5-epimerase), *clbI* (colibactin polyketide synthase ClbI), *clbH* (colibactin non-ribosomal peptide synthetase clbH), *VasK* (type VI secretion protein VasK) and *clbC* (colibactin polyketide synthase ClbC). All data are shown as means ± SD. (C) The correlation coefficient (*R*^2^) between the two data sets of bEnd.3 cells. The *x*-axis represents the log_2_ fold change in expression determined by qRT-PCR and the *y*-axis represents the log_2_ fold change in expression determined by RNA-seq. (D) The correlation coefficient (*R*^2^) between the two data sets of APEC strain. The *x*-axis represents the log_2_ fold change in expression determined by qRT-PCR and the *y*-axis represents the log_2_ fold change in expression determined by RNA-seq.

To confirm the results of the RNA-seq analysis, 17 highly expressed DEGs were randomly selected for further validation using qRT-PCR (details in Table S1). The trends of up- and down-regulation for 16 of the genes were consistent with the results of the Illumina sequencing analysis, except the *Vask* gene (Figs. 1A and 1B). In addition, the Pearson correlation coefficients (*R*^2^) of bEnd.3 cells and APEC strain were 0.954 and 0.876, respectively, indicating that the results of both techniques correlated strongly (Figs. 1C and 1D), thus confirming the reliability and accuracy of the transcriptome analysis.

### Analysis of changes in the bEnd.3 cells transcriptome during infection

**Figure 2:**
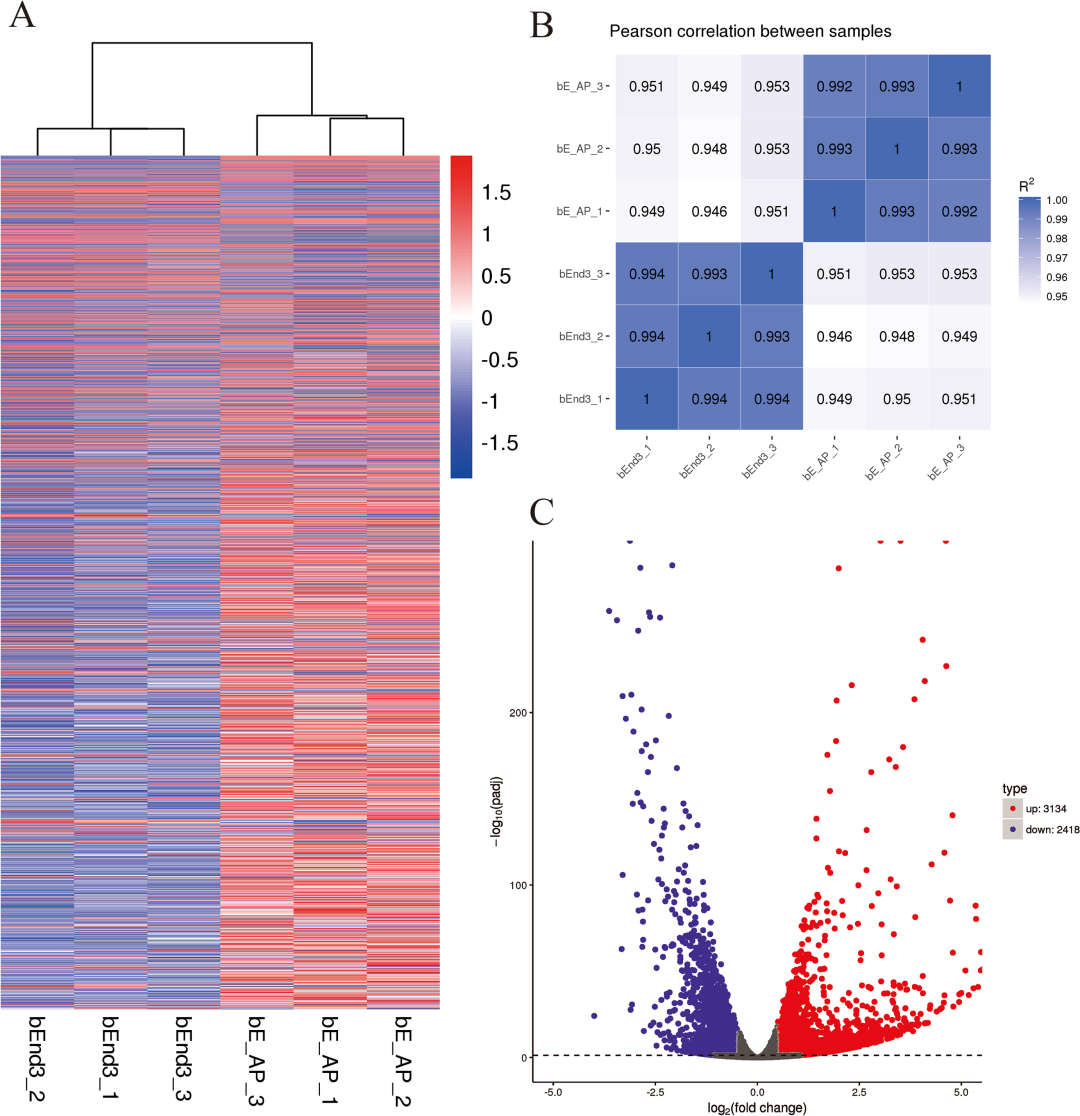
Differential expression overview profiles of bEnd.3 cells transcriptome data. (A) Heat maps of bEnd.3 gene expression during infection or mock infection samples. The read counts of each cellular mRNA were normalized by the sum of the total reads. Colors from white to red indicate upregulated cellular genes; colors from white to blue indicate downregulated cellular genes. (B) Pearson correlation between infection and mock infection samples. (C) Volcano plot of *P* values as a function of weighted fold change for mRNAs in infected and control groups. The vertical dotted line delimits up- and down-regulation. Red plots represent significant upregulated and green plots represent significant downregulated. (|log_2_ fold change| of ≥0.5, corrected *P* < 0.05).

The hierarchical clustering (Fig. 2A) and RNA-seq sample Pearson correlation analysis (Fig. 2B) of gene expression datasets from the infected and uninfected bEnd.3 cells demonstrated high reproducibility within group. The DESeq2 R package identified 5,552 DEGs between the uninfected and infected bEnd.3 cells, among which 3,134 were upregulated and 2,418 were downregulated in response to infection (Fig. 2C; |log_2_-fold change| ≥ 0.5 and adjusted *P* value < 0.05).

**Figure 3:**
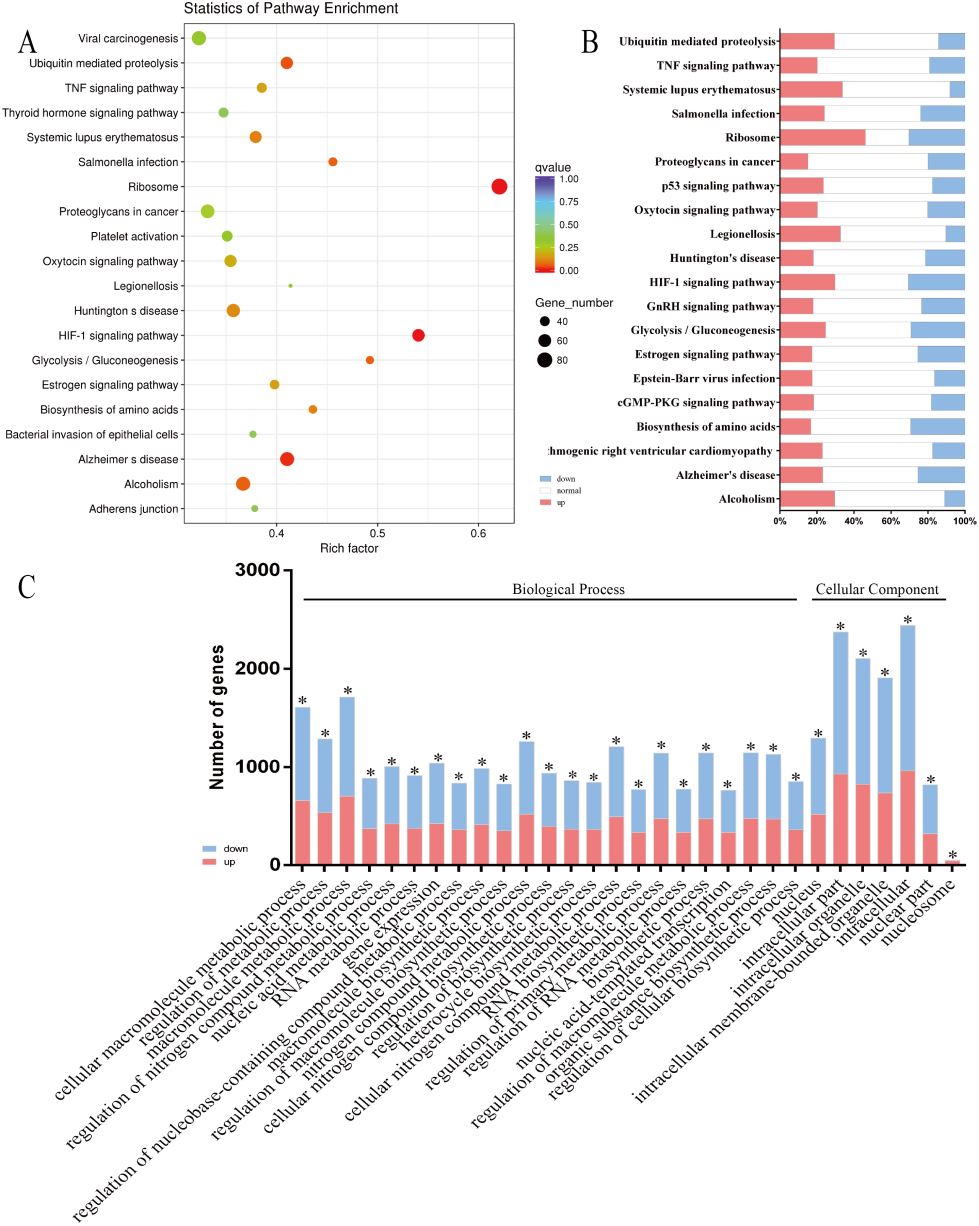
Enrichment analysis of KEGG pathways and GO terms for DEGs in bEnd.3 cells. (A and B) The top 20 enriched KEGG pathways were classified as human disease (9/20, Salmonella infection and Legionellosis etc), endocrine system (4/20, Estrogen signaling pathway and Oxytocin signaling pathway etc), signal transduction (2/20, HIF-1 signaling pathway, TNF signaling pathway) and other pathways. The size of each circle represents the number of DEGs in each pahway (larger circles represent more DEGs) and the color represents the corrected *P* value of each pathway. Red bars represent significant upregulated, blue bars represent significant downregulated and white bars represent no expression or no significant expression. (C) In the top 30 enriched GO terms, most terms (23/30) were classified as biological process, seven of them were belonged to cellular component.Red bars represent significant upregulated and blue bars represent significant downregulated (|log_2_ fold change| ≥0.5, *corrected *P* < 0.05).

Functional classification of the DEGs using KEGG pathway enrichment analysis revealed their association with 273 pathways, indicating that many host genes, whose expression changed in response to infection, were enriched in signal transduction and immune response. The top 20 pathways are shown in Figs. 3A and 3B. The DEGs were also annotated by GO enrichment analysis using the GOseq R package, which showed that the DEGs were enriched in 12,833 GO terms, including 9,505 biological process terms, 1,158 cellular component terms and 2,152 molecular function terms. The majority of the top 30 enriched GO terms (23/30) were classified as biological processes, details of which are shown in Fig. 3C. This study focused on the interesting changes in the bEnd.3 cells, including DEGs responsible for altering the integrity of host cell junctional complexes, actin cytoskeletal rearrangements, extracellular matrix (ECM) degradation, immune activation, and inflammatory responses (Table 2; Table S2).

**Table 2:** DEGs of bEnd.3 cells between the two groups. Different expression genes of bEnd.3 cells between the infection and mock-infection group.

	Components	Genes	log_2_	padj	Description
			Fold change		
DEGs related to cell junctional complexes					
Tight junctions (TJs)	Claudins	*Cldn5*	−0.51506	0.000166	claudin 5
Adherens junctions (AJs)	Cadherin	*Cdh5*	−0.73355	3.27E−19	cadherin 5
		*Cdh24*	−0.78667	0.041049	cadherin-like 24
	nectin	*Nectin1*	−1.7466	1.22E−50	nectin cell adhesion molecule 1
		*Nectin2*	−0.53373	0.00102	nectin cell adhesion molecule 2
		*Nectin3*	0.59157	3.14E−07	nectin cell adhesion molecule 3
DEGs related to actin cytoskeletal rearrangements					
Regulation of actin cytoskeleton		*Cfl1*	−0.54554	3.27E−10	cofilin 1, non-muscle
		*Actn1*	−0.60337	2.06E−09	actinin, alpha 1
		*Limk1*	−0.6284	0.001253	LIM-domain containing, protein kinase
		*Pxn*	−0.83324	3.61E−10	paxillin
		*Actn4*	−1.6743	1.54E−27	actinin alpha 4
		*Actb*	2.0004	1.10E−136	actin, beta
		*Itgav*	0.78575	1.76E−21	integrin alpha V
		*Rock1*	0.61447	2.59E−12	Rho-associated coiled-coil containing protein kinase 1
		*Rock2*	0.50308	2.05E−09	Rho-associated coiled-coil containing protein kinase 2
DEGs of immune activation and inflammatory response					
Pattern recognition receptors (PRRs)		*Tlr13*	1.2499	6.48E−16	toll-like receptor 13
		*Tlr4*	0.82039	2.48E−17	toll-like receptor 4
Complement system		*C3*	−0.58759	0.005068	complement component 3
		*C3ar1*	0.74408	2.90E−05	complement component 3a receptor 1
		*Cfp*	−0.99477	3.44E−05	complement factor properdin
		*Masp1*	3.9332	0.00077	mannan-binding lectin serine peptidase 1
Chemokines	C subfamiliy	Xcr1	1.749	6.96E−06	chemokine (C motif) receptor 1
	C–C subfamiliy	*Ccl2*	0.61885	0.000328	chemokine (C–C motif) ligand 2
		*Ccrl2*	0.72992	0.03855	chemokine (C–C motif) receptor-like 2
	C–X3–C subfamiliy	*Cx3cl1*	−0.54859	0.000456	chemokine (C–X3–C motif) ligand 1
	C–X–C subfamiliy	*Cxcl1*	1.7688	2.22E−06	chemokine (C–X–C motif) ligand 1
		*Cxcl16*	1.4227	0.000779	chemokine (C–X–C motif) ligand 16
		*Cxcl2*	1.7977	0.00055	chemokine (C–X–C motif) ligand 2

The BBB is a highly specialized structural and biochemical barrier; its properties are primarily determined by junctional complexes between the endothelial cells (ECs), comprising of adherens junctions (AJs) and tight junctions (TJs). In this study, genes encoding AJs (e.g., *Cdh5, Cdh24, Pcdh1, Pcdhgc3, Nectin1* and *Nectin2*), which were involved in supporting cadherin association and regulating out-in signaling processes, were downregulated, but *Nectin3* was upregulated in *E. coli*-infected bEnd.3 cells. Moreover, genes encoding TJs (e.g., *Cldn5, Tjap1, Actn1* and *Actn4*), which are involved in sealing the interendothelial cleft, were downregulated in the infected cells. These findings indicated that the structural integrity, permeability and paracellular barriers of the BBB were destroyed during infection.

Previously, it has been shown that the actin cytoskeleton was rearranged and the ECM, with related receptors, were closely regulated when NMEC traversed the BBB (Kim, Kang & Kim, 2005; Kim, 2002). In the present study, some DEGs (*Rock1*, *Rock2*, *Vav3*, *Itgav*, *Lamc2*, *Sdc4*, *Gp1ba*, and *Thbs1*) related to actin arrangement and the ECM were upregulated, while other DEGs with similar functions (*Actn4*, *Actn1*, *Itgb4, Pxn*, *Cfl1*, *Wasf1*, *Wasl*, *Dag1*, *Col5a3*, *Col5a1*, *Itga5*, *Col27a1*, *Lama5*, *Itgb4*, *Fn1*, *Agrn*, *Comp*, *Col1a1* and *Hspg2*) were downregulated. These findings suggested an increase in Rho/ROCK pathway activation, F-actin cytoskeleton rearrangement, and BBB permeability.

Inflammation is a hallmark of bacterial meningitis and is mediated mainly by cytokines and chemokines, which occurs in response to bacteria or their products. Additionally, *Tlr4*, *Tlr13, Cxcl2*, *Cxcl1*, *Xcr1*, *Cxcl16*, *Nfkbia*, *Tnfaip3*, *Il6, Casp12*, *Nod2*, *Ccl2*, *Vcam1*, *Ncf2*, *Cfd*, *Cd46* and *C3ar1* were upregulated and *C3* was downregulated in the infection group, which suggested that the permeability of the BBB increased and the recruitment of monocytes, neutrophils, T cells, and natural killer cells was enhanced during the infection process.

### *E. coli* transcriptome changes during bEnd.3 cells infection

**Figure 4:**
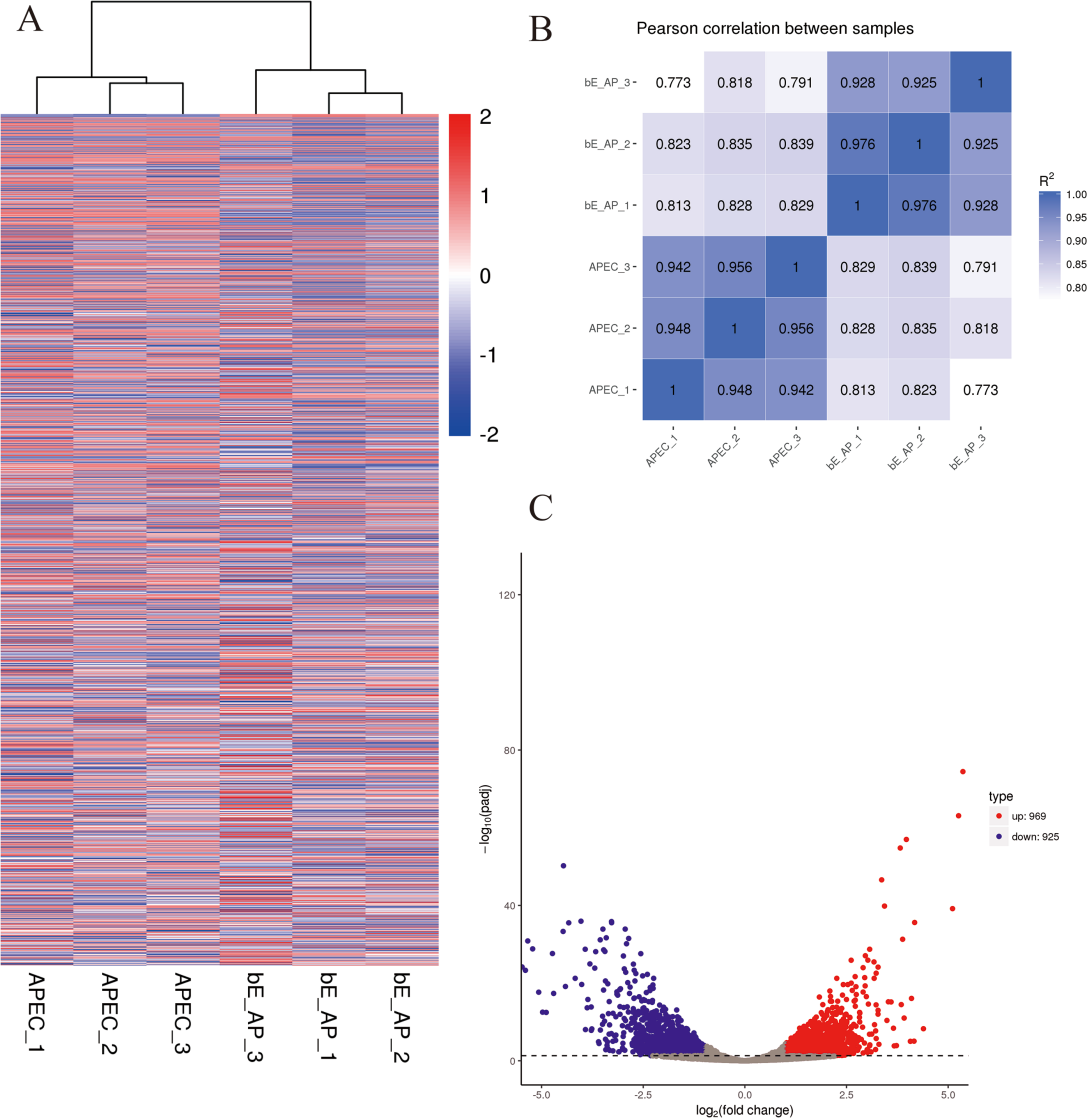
Differential expression overview profiles of APEC strain transcriptome data. (A) Heat maps of APEC strain gene expression during infection or control samples. The read counts of each cellular mRNA were normalized by the sum of the total reads. Colors from white to red indicate upregulated cellular genes; colors from white to blue indicate downregulated cellular genes. (B) Pearson correlation between infection and control samples. (C) Volcano plot of *P*-values as a function of weighted fold change for mRNAs in infection and control groups. The vertical dotted line delimits up- and down-regulation. Red plots represent significant upregulated and green plots represent significant downregulated (|log_2_ fold change| ≥1, corrected *P* < 0.05).

The hierarchical clustering (Fig. 4A) and RNA-seq sample Pearson correlation analysis (Fig. 4B) of gene expression datasets demonstrated high reproducibility within group from the infected and mock-infected bEnd.3 cells. The DESeq2 R package identified 1,894 DEGs between the two infection conditions, including 969 upregulated and 925 downregulated genes (Fig. 4C, |log_2_ fold change| ≥ 1 and adjusted *P-*value < 0.05).

**Figure 5:**
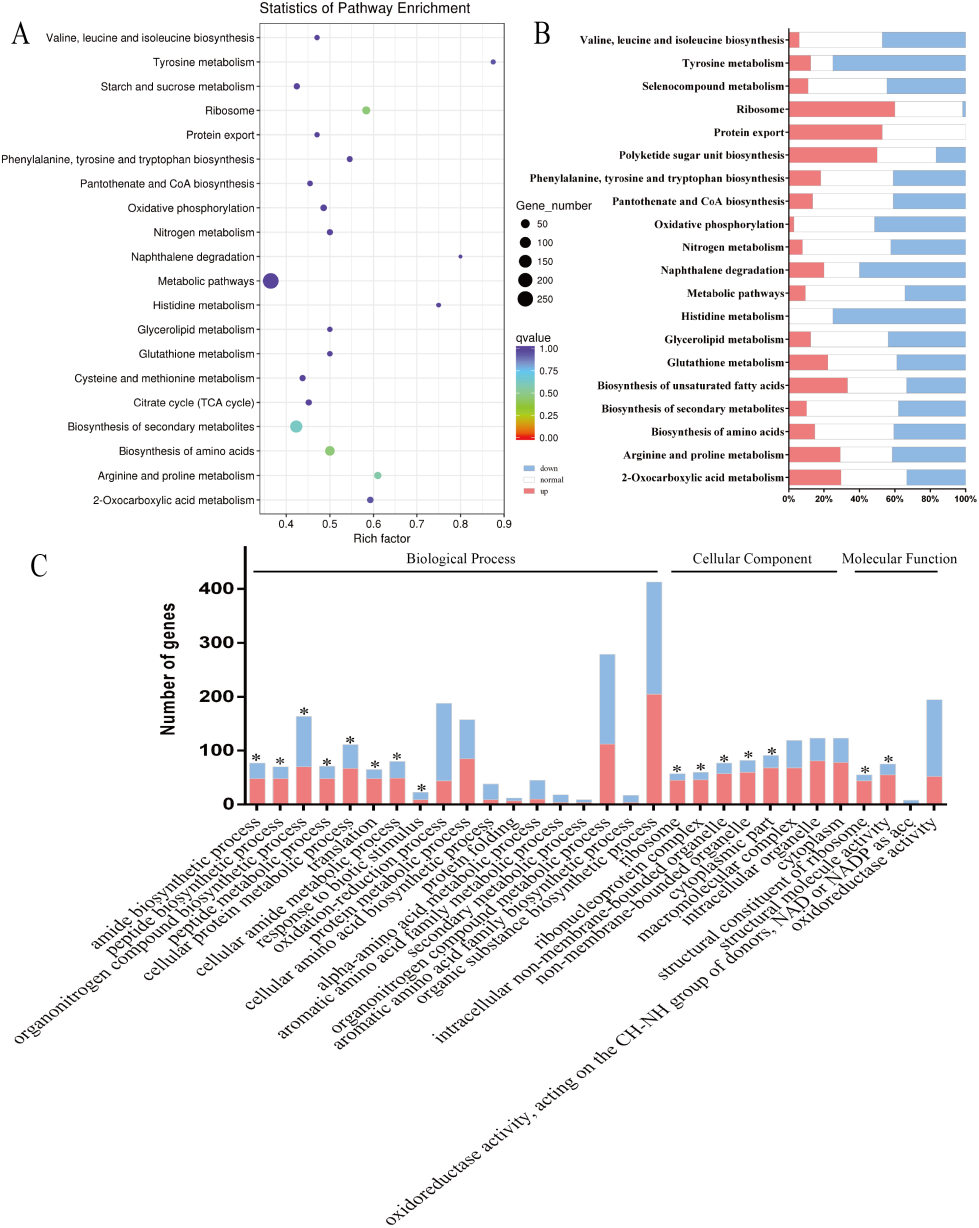
Enrichment analysis of KEGG pathways and GO terms for DEGs in APEC strain. (A and B) The top 20 enriched KEGG pathways were classified as amino acid metabolism (7/20, Valine, leucine and isoleucine biosynthesis and Selenocompound metabolism, etc.), lipid metabolism (2/20, Biosynthesis of unsaturated fatty acids and Glycerolipid metabolism), energy metabolism (2/20, Nitrogen metabolism and Oxidative phosphorylation) and othe pathways. The size of each circle represents the number of DEGs in each pahway (larger circles represent more DEGs) and the color represents the corrected *P* value of each pathway. Red bars represent significant upregulated, blue bars represent significant downregulated and white bars represent no expression or no significant expression. (C) In the top 30 enriched GO terms, most terms (18/30) were classified as biological process, eight of them belonged to cellular component and four of them were classified as molecular function. Red bars represent significant upregulated and blue bars represent significant downregulated (|log_2_ fold change| ≥1, *corrected *P* < 0.05).

KEGG pathway enrichment analysis was used to functionally classify the DEGs for 88 pathways, revealing that many *E. coli* genes were enriched in amino acid and energy metabolism when APEC was cultured with the cells. The top 20 pathways are shown in Figs. 5A and 5B. The DEGs were also annotated by GO enrichment analysis using the GOseq R package, and enriched for 2,482 GO terms, including 1,376 biological process terms, 311 cellular component terms and 795 molecular function terms. The majority of the top 30 enriched GO terms (18/30) were classified as biological processes, which are shown in detail in Fig. 5C. In this study, we also identified several interesting changes in *E. coli*, particularly in the DEGs related to virulence factors, protein export systems and amino acid metabolism (Table 3; Table S3).

**Table 3:** DEGs of APEC strain between two groups. Different expression genes of APEC strain between the infection and mock-infection group.

	Components	Gene name	log_2_ Fold Change	padj	Description
Virulence factors related to meningitis					
Outer membrane protein	CXG97_RS20855	–	−2.3638	3.43E−06	membrane protein
	CXG97_RS13750	–	−1.8361	0.0083262	membrane protein
	CXG97_RS15480	–	−1.7665	0.014095	membrane protein
	CXG97_RS10635	–	−1.1948	0.039525	membrane protein
	CXG97_RS12085	–	3.121	3.42E−09	membrane protein
	CXG97_RS18795	–	1.5797	0.003432	membrane protein
	CXG97_RS10035	–	1.1784	0.032157	membrane protein
	CXG97_RS08525	–	1.3149	0.015745	autotransported outer membrane protein involved in cell adhesion
	CXG97_RS01930	–	1.7816	0.0014647	autotransporter outer membrane beta-barrel domain-containing protein
	CXG97_RS01635	–	1.4776	0.0098823	autotransporter outer membrane beta-barrel domain-containing protein
	CXG97_RS09010	–	1.6293	0.049761	fimbrial biogenesis outer membrane usher protein
	CXG97_RS18505	–	1.3315	0.045966	fimbrial biogenesis outer membrane usher protein
	CXG97_RS09580	*slyB*	1.3411	0.0099186	outer membrane lipoprotein SlyB
	CXG97_RS15430	–	1.5911	0.0023044	outer membrane protein assembly factor BamD
	CXG97_RS15540	–	1.2863	0.015568	outer membrane protein assembly factor BamE
fimbrial	CXG97_RS01490	–	−3.3352	2.07E−05	fimbrial chaperone EcpB
	CXG97_RS17665	–	−1.2869	0.0229	fimbrial protein SteB
	CXG97_RS25945	*fimA*	−1.1275	0.041784	type 1 fimbriae major subunit
	CXG97_RS13610	–	−1.7529	0.002143	flagella biosynthesis regulator Flk
	CXG97_RS25950	–	−2.2246	0.00011843	fimbrin fimI
	CXG97_RS09010	–	1.6293	0.049761	fimbrial biogenesis outer membrane usher protein
	CXG97_RS09025	–	2.7839	2.36E−07	fimbrial chaperone protein FimC
fimbrial	CXG97_RS09015	–	1.7675	0.0015729	fimbrial chaperone protein FimC
	CXG97_RS17675	–	1.996	8.94E−05	type 1 fimbrial protein
	CXG97_RS20510	–	1.2399	0.02421	type 1 fimbrial protein
	CXG97_RS09020	–	2.3148	5.86E−06	Fml fimbriae subunit
flagellin	CXG97_RS11050	–	1.2589	0.039065	flagellin FliC
LPS biosynthesis	CXG97_RS21640	–	2.4423	3.2651E−06	ligase
	CXG97_RS21660	–	1.9943	0.00014874	LPS 1%2C2-glucosyltransferase
	CXG97_RS21655	–	2.0378	0.00015131	LPS core heptose(II) kinase RfaY
	CXG97_RS21665	–	1.8557	0.00042876	lipopolysaccharide 3-alpha-galactosyltransferase
	CXG97_RS06255	–	−1.3255	0.017077	lipid A biosynthesis lauroyl acyltransferase
	CXG97_RS21670	–	1.2497	0.019185	lipopolysaccharide core heptose(I) kinase RfaP
	CXG97_RS21630	–	−1.7497	0.038733	ADP-heptose–LPS heptosyltransferase
Proteins export and amino acid metabolism					
Protein export	CXG97_RS19815	*SecY*	2.0511	4.25E−05	protein translocase subunit SecY
	CXG97_RS23005	*tatC*	1.9541	0.00021961	twin-arginine translocase subunit TatC
	CXG97_RS02115	*YajC*	1.7879	0.00069815	preprotein translocase subunit YajC
	CXG97_RS22995	*TatA*	1.3288	0.012952	twin-arginine translocase subunit TatA
	CXG97_RS23000	*TatB*	1.2944	0.014643	twin-arginine translocase subunit TatB
	CXG97_RS02125	*SecF*	2.2484	0.020017	protein translocase subunit SecF
	CXG97_RS21575	*SecB*	1.237	0.025291	protein-export protein SecB
	CXG97_RS19155	*SecG*	1.1816	0.027567	protein-export membrane protein SecG
Arginine and proline metabolism	CXG97_RS23620	*argB*	3.3547	1.35E−05	acetylglutamate kinase
	CXG97_RS17080	*arcC*	1.6337	0.008478	carbamate kinase
	CXG97_RS08705	*patD*	1.3149	0.037641	gamma-aminobutyraldehyde dehydrogenase
	CXG97_RS25795	*argF*	8.588	1.37E−24	ornithine carbamoyltransferase
	CXG97_RS03780	*speF*	1.5823	0.034956	ornithine decarboxylase SpeF
	CXG97_RS06060	*putA*	−3.5858	0.005959	bifunctional proline dehydrogenase/L-glutamate gamma-semialdehyde dehydrogenase PutA
	CXG97_RS10130	*astB*	−4.4096	0.006318	succinylarginine dihydrolase
	CXG97_RS10135	*astD*	−5.5193	0.000153	N-succinylglutamate 5-semialdehyde dehydrogenase
	CXG97_RS17430	*speB*	−1.8935	0.000611	agmatinase
	CXG97_RS23160	*glnA*	−1.458	0.009095	glutamate–ammonia ligase

A critical step in the development of meningitis is the adhesion and invasion of ECs. Therefore, virulence factors related to fimbriae, flagella, outer membrane proteins, and lipoproteins are highly important for allowing pathogenic *E. coli* to resist blood flow and cross the BBB.

The RNA-seq data showed that the expression of seven genes related to outer membrane proteins (e.g., CXG97_RS09580, CXG97_RS01930), eight fimbrial genes (e.g., CXG97_RS09010, CXG97_RS09025), one flagellin gene (CXG97_RS11050), two pilus genes (CXG97_RS17695 and CXG97_RS17680), and one lipoprotein gene (CXG97_RS19095) increased significantly during infection, whereas the expression of nine fimbrial genes (e.g., CXG97_RS01490, CXG97_RS25950) decreased during infection (Table 3; Table S3).

Lipopolysaccharide (LPS) is produced by most Gram-negative bacteria and can activate the host immune system via TLR4. RNA-seq data revealed that eight DEGs were enriched in the LPS biosynthesis pathway, five of which (e.g., CXG97_RS21640, CXG97_RS21660) were upregulated in the infection group. Colicins are class III bacteriocins, which are produced during nutrient or oxygen stress and regulated by the SOS response (Smarda & Smajs, 1998). The expression of six genes related to colicin biosynthesis and transport (e.g., CXG97_RS26815, CXG97_RS27750) increased significantly during infection. In addition, thirteen colibactin genes (e.g., CXG97_RS11550, CXG97_RS11545), which induce chromosomal instability and DNA damage in eukaryotic cells and lead to EC senescence and immune cell apoptosis, were downregulated during infection, while only one colibactin gene (CXG97_RS11495) was upregulated. The type VI secretion system (T6SS) contributes to the pathogenicity of bacteria (Zhou et al., 2012) and bacteria–bacteria interactions (Basler, Ho & Mekalanos, 2013). Four T6SS genes (e.g., CXG97_RS01210, CXG97_RS01220) were downregulated during infection in the present study. ABC transporters are essential bacterial virulence factors, which play roles in the secretion of toxins and antimicrobial agents, and are associated with physiological processes (Davidson & Chen, 2004). In RNA-seq data, numerous DEGs were enriched in the ABC transporter pathway, with CXG97_RS18120, CXG97_RS13545, CXG97_RS23390 and CXG97_RS05305 being the most significantly upregulated genes in the APEC strain cultured with cells. These results suggested that the occurrence of meningitis was related to synergistic effects of many virulence factors.

Compared to the negative control, the prokaryotic protein export pathway was one of the most enriched *E. coli* KEGG pathways, which is composed of the general secretory system (Sec system), twin-arginine translocase (Tat) system, and a single peptide. The Sec and Tat systems are responsible for the transport of unstable or unfolded bacterial structural proteins to the periplasm or cytoplasmic membrane. Some genes (*secYFBG*, *tatABC*, *yajc* and *lepB*) from three parts of this pathway were upregulated in the infected groups, which suggested that protein secretion may be increased in the infected group. Moreover, several DEGs related to amino acid metabolism in APEC changed in response to bEnd.3 cells, as compared to those cultured in DMEM. The majority of these genes, which were involved in arginine and proline metabolism (*argB, astDB*, *speBF* and *patD*), histidine metabolism (*hisHDFAF*), and valine, leucine and isoleucine biosynthesis (*leuCB* and *ilvA*), were downregulated in the infected samples, suggesting that APEC may encounter a difficult and complex nutritional environment during the infection process.

## Discussion

Bacterial meningitis is an inflammatory disease of the CNS, which not only causes high morbidity and mortality but also leaves survivors with long-term neurological sequelae. To infect the CNS, bacteria must interact with and cross the BBB via a critical step involving the adherence and invasion of BMECs, an important component of the BBB. In the present study, we used RNA-seq to measure genome-wide transcriptional changes in both APEC and bEnd.3 cells, including cell junctional complexes, cell signaling, inflammatory responses, bacterial adhesion and invasion factors and metabolic competition for similar nutritional substrates. The findings of the present study may contribute toward an improved understanding of the microbe-cell interaction during the invasion process.

TJ and AJ proteins can form junctional complexes and thus play important roles in maintaining the integrity of the BBB (Tietz & Engelhardt, 2015). We found that major components of TJs and AJs, such as *Cldn5* and *Cdh5*, were downregulated in infected cells. *Cdh5* is involved in neuroinflammation development, BBB dysregulation (Gijbels et al., 1990), and leukocyte transmigration in vitro (Orsenigo et al., 2012), and affects the expression of other TJ and AJ proteins (Dejana & Vestweber, 2013; Orsenigo et al., 2012). Conversely, *Cldn5* highly expressed in ECs in the CNS (Daneman et al., 2010; Ohtsuki et al., 2008), which plays a key role in the paracellular barrier and forms mechanical links to maintain the structural integrity and high electrical resistance of vasculature (Abbott et al., 2010). The changes in *Cdh5 and Cldn5* expression observed in the present study are consistent with the previous findings reported in a *Staphylococcus aureus* and *group B Streptococcus* model of meningitis (Kim et al., 2015; McLoughlin et al., 2017). In addition, *Cdh5* controls *Cldn5* by triggering its transcriptional repression via *FoxO1* and β*-catenin* (Taddei et al., 2008). Transcriptome data in the present study revealed a significant decrease in *FoxO1* expression during infection but only a slight increase in β*-catenin* expression, which may be related to the up-regulation of β-catenin protein levels and the activation of Wnt/β-catenin signaling by LPS (Xing et al., 2019). Moreover, Wnt/β-catenin signaling was shown to be involved in BBB development, where its blockade decreased *Cdh5* expression in primary ECs of newborn mouse brain but not in that of the adult (Hubner et al., 2018). The results of the present study as well as previous studies (Kim et al., 2015; McLoughlin et al., 2017) suggest that *Cdh5* and *Cldn5* are major determinants of BBB deterioration during infection, while Wnt/β-catenin signaling may contribute to the maintenance of BBB integrity. However, further studies are required to investigate the complex relationship between *Cdh5*, *Cldn5* and Wnt/β-catenin signaling during the development of *E. coli* meningitis.

As a dual (physical and immunological) barrier, the BBB is also a central determinant of protective homeostatic surveillance during CNS infections (Klein & Hunter, 2017). CNS ECs are semiprofessional antigen-presenting cells that present antigens to T cells and regulate the multistep cascade for immune cell trafficking into the CNS (Meyer, Martin-Blondel & Liblau, 2017). In the present study, *Tlr4* and *Tlr13* were upregulated in infected cells. However, a previous study on mouse meningitis induced by *E. coli* showed that TLRs were activated in brain tissues, with elevated *Tlr2, Tlr4* and *Tlr7* expression (Bottcher et al., 2003). TLR activation has also been shown to modulate microvascular EC permeability and the expression of coagulation pathway intermediaries (Khakpour, Wilhelmsen & Hellman, 2015). *Tlr13* was highly expressed in almost all mouse CNS cell types and specifically detected 23S ribosomal RNA from *E. coli* (Li & Chen, 2012). Recent studies have identified that the recognition of *E. coli* mRNA stimulated helper T cell differentiation, promoted vaccine responses, and helped to distinguish between live and dead microbes (Sander et al., 2011; Ugolini et al., 2018). To our knowledge, this is the first study to report the high expression of *Tlr13* in *E. coli* meningitis model in vitro. However, further investigations are necessary to elucidate the specific microbial components that activate Tlr13 in *E. coli* meningitis and reveal the details of the related pathways involved in infection. Moreover, future studies should investigate TLR inhibitors as potential targets to prevent serious meningitis-related complications.

Following inflammatory activation, host cells release cytokines and chemokines to maintain immune surveillance, facilitate leukocyte traffic, and recruit other inflammatory factors (Takeshita & Ransohoff, 2012). In the present study, *Cxcl1, Cxcl2* and *Cxcl16* were significantly upregulated in infected cells. A similar innate immune response was previously observed in different bacterial meningitis as well. *Cxcl1*, *Cxcl2* and *CXCL16* were associated with the migration of immune cells to sites of inflammation, matrix metalloproteinase activity, increased cell–cell adhesion, NF-кB-dependent cell proliferation, and proinflammatory gene transcription (Chandrasekar, Bysani & Mummidi, 2004; Girbl et al., 2018; Griffith, Sokol & Luster, 2014; Hofnagel et al., 2011; Semple, Kossmann & Morganti-Kossmann, 2010; Van Der Voort et al., 2005). In addition, *Cxcl1* and *Cxcl2* have been shown to alter human BMEC permeability and disrupt EC junctions during the migration of neutrophils and monocytes (Girbl et al., 2018; Zhang et al., 2013).

The mechanism of *E. coli* pathogenesis involves complex patterns of adhesion, protein export into host cells, changes in signaling mechanisms, impaired immune responses with colonization, disrupted membrane potential, and cytoskeletal manipulation (Bhavsar, Guttman & Finlay, 2007; Hornef et al., 2002; Kim et al., 2010). In the present study, many bacterial DEGs related to fimbrial and flagellin components were upregulated in the infection group. Type 1 fimbriae are mainly formed by FimAGHF proteins and mediate the mannose-sensitive adhesion of *E. coli* to various eukaryotic cells (Hanson & Brinton, 1988; Klemm, 1984; Teng et al., 2005). Conversely, the expression of S fimbriae in *E. coli* promoted adhesion to cow, human, and rat BMECs but not the systemic vascular endothelium (Prasadarao, Wass & Kim, 1997). Moreover, it has been shown that flagella, the locomotive organelles of bacteria, are an association factor rather than an invasion factor in human BMECs (Parthasarathy, Yao & Kim, 2007). The results of the present study also indicated that fimbrial and flagellin components were highly important virulence factors of the APEC XM strain for BBB attachment and invasion.

*E. coli* exerts physiological or pathogenic functions by exporting proteins via eight different systems (Crane & Randall, 2017). In the present study, many DEGs (*secYFBG* and *LepB)* related to the Sec system were upregulated in the infection group. *SecB* plays a crucial role as a chaperone during protein secretion by binding to precursors and delivering them to the membrane for translocation. Previous studies have shown that many virulence factors, such as P pilus, type 1 pilus, curli, OmpT and OmpA, were secreted into the extracellular environment or localized in the outer membrane by the SecYEG complex or SecB chaperone (Baars et al., 2006; Stathopoulos et al., 2000; Stones & Krachler, 2015). Recently, *LepB* has been proved to be a potential target for an attractive new antibacterial agent due to its crucial role in the Sec pathway; LepB inhibition leads to preprotein accumulation at the phospholipid bilayer and thus cell death (De Rosa et al., 2017; Ferrandez & Condemine, 2008). On the basis of these results, Sec pathway-mediated secretion may play an important role in bacterial pathogenesis. Thus, Sec pathway-associated proteins could be potential antibiotic drug targets for the prevention and treatment of meningitis. Another major component of the protein export pathway is the Tat system. In the present study, the three primary components of the Tat system (*tatABC)* were upregulated in the infected group. The Tat system was shown to take part in the development of bacteremia as well as the production of Shiga toxin 1 (Stx1) and H7 flagellin (Siddiqui, Beattie & Khan, 2012). Therefore, these results suggest that the Tat system may have a potential role in virulence during meningitis.

In addition to the deterioration of physical and immunological barrier functions, host cells and pathogens fiercely competed for nutrition. Indeed, the metabolic competition between the host and bacteria could influence both bacterial virulence and host responses, which determine the outcome of infection (Olive & Sassetti, 2016). In the present study, we identified a series of DEGs related to arginine and proline metabolism, which are particularly important nutrients for the host–pathogen interaction. Arginine is the unique substrate for nitric oxide synthase (NOS) to generate nitric oxide (NO) (Palmer, Ashton & Moncada, 1988), which has a variety of physiological functions, including vasodilation, leukocyte activation, and killing of virus and bacteria in diseases (Chicoine et al., 2004). In the present study, four host genes (*Gm15587*, *Nos2*, *Pycr1*, *Arg2*) were enriched in this metabolic pathway, two of which (*Nos2*, *Pycr1*) were downregulated in the infection group. *Nos2* encoded a NOS and contributed to BBB breakdown and thus early mortality in murine *Streptococcus pneumoniae* meningitis (Yau et al., 2016). Previous studies showed that *E. coli* utilized arginine via the arginine decarboxylase and the arginine succinyltransferase pathway to produce polyamines (putrescine, spermidine, and spermine) for proliferation or survival in an acidic environment (Schneider, Kiupakis & Reitzer, 1998; Stancik et al., 2002). Since arginase and iNOS have the same substrate, *E. coli* may exploit their competition to block NO production and thus avoid being killed by NO in a similar manner to other pathogens (Bronte & Zanovello, 2005; Eckmann et al., 2000; Talaue et al., 2006). In our RNA-seq data set, some DEGs related to arginine and proline metabolism (*argBF, arcC, patD*, and *speF*) were upregulated in the infection group, while others (*putA*, *astBD*, *speB* and *glnA*) were downregulated. On the basis of these results, arginine/ornithine ABC transporters may be an effective target for preventing meningitis. Furthermore, the simultaneous changes in arginine and proline metabolism in the host and microbe may provide novel insights into nutrient and metabolite competition that occurs during meningitis development. Further experiments are required to explore this field and discover the molecular mechanisms underlying the host–pathogen relationship during infection.

## Conclusion

This study provided a comprehensive overview of the transcriptomic changes that occurred when APEC infected the bEnd.3 cells. APEC may exploit the degradation of cell junctional connections to invade the BBB and secrete virulence factors to promote bacterial infection. Meanwhile, bEnd.3 cells resisted the bacterial infection via immune activation and inflammatory response. Therefore, this study provides insights into the process of bacterial invasion and the subsequent host defense mechanism, which can be used as reference for further investigations in this field.

## Supplemental Information

10.7717/peerj.9172/supp-1 Supplemental Information 1 Bacterial adherence and invasion of bEnd.3 cells. Adherence to and invasion of bEnd.3 cells by APEC strain (at MOI of 100). Data are means + standard errors of three independent experiments, each performed in triplicate. An asterisk indicates that the adherence and invasion values of others’ time points were significantly higher than the values of the first one hour at *P*-value < 0.01.

10.7717/peerj.9172/supp-2 Supplemental Information 2 bEnd.3 cell and APEC gene-specific primers for qRT-PCR.

10.7717/peerj.9172/supp-3 Supplemental Information 3 DEGs of bEnd.3 cells between the two groups.

10.7717/peerj.9172/supp-4 Supplemental Information 4 DEGs of APEC strain between two groups.

## Additional Information and Declarations

### Data Availability

The following information was supplied regarding data availability: The APEC RNA-sequences and bEnd.3 cells RNA-sequences are available at SRA: (PRJNA612631) SRR11307869, SRR11307868, SRR11307867, SRR11307872, SRR11307871 and SRR11307870; (PRJNA612931) SRR11318238, SRR11318235, SRR11318236, SRR11318234, SRR11318233 and SRR11318237. The data is also available at Zenodo: wang peili. (2019). Raw data of RNA-seq and IGV -2 [Data set]. Zenodo. DOI 10.5281/zenodo.3689240. and Wang peili. (2019). Raw data of RNA-seq and IGV -1 [Data set]. Zenodo. DOI 10.5281/zenodo.3672826.
