# Supplementary figures and images for: Transcriptome profiling of avian pathogenic Escherichia coli and the mouse microvascular endothelial cell line bEnd.3 during interaction
Source: PeerJ. 2020 May 21;8:e9172. doi: 10.7717/peerj.9172 (PMC7246031; doi:10.7717/peerj.9172)

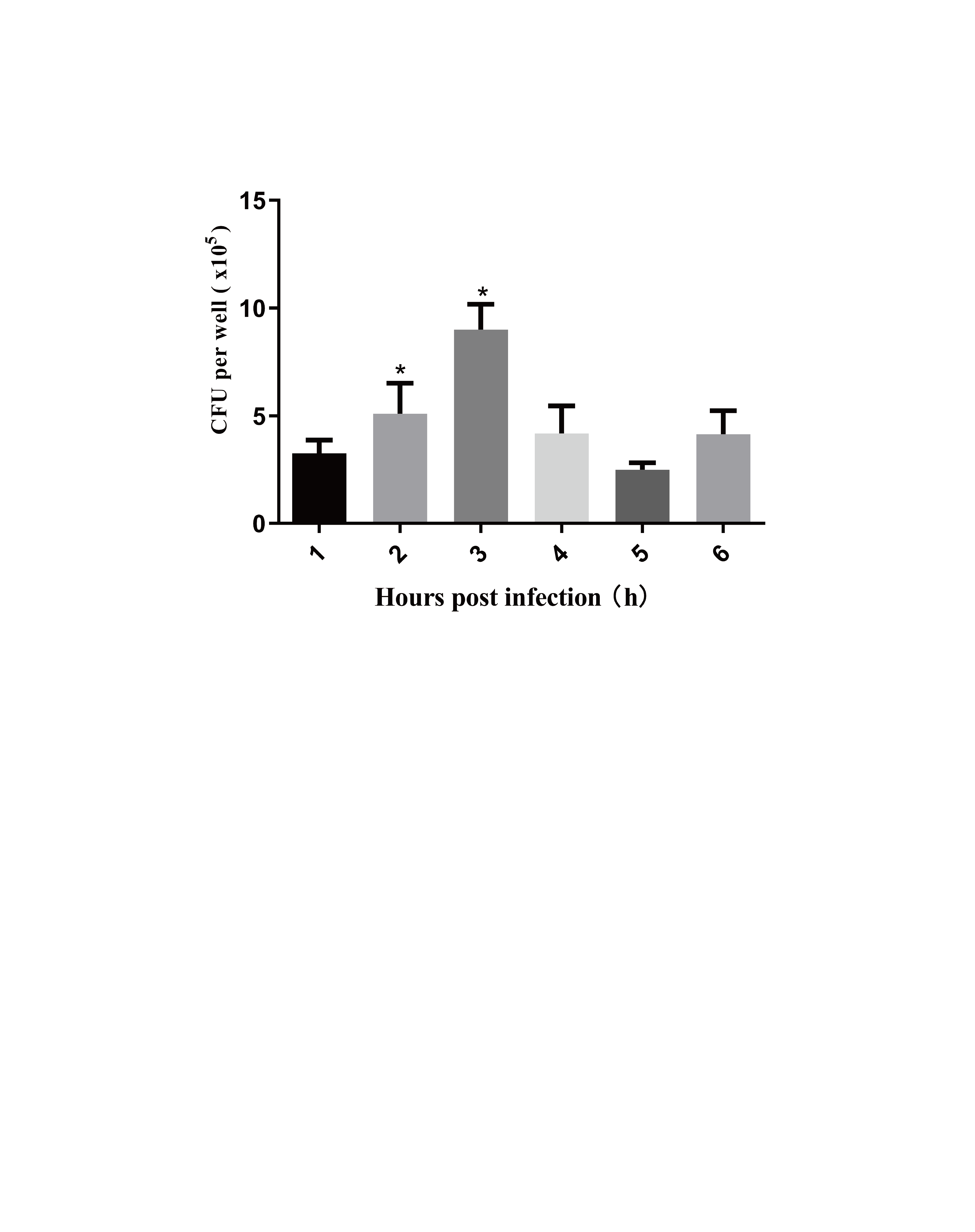

Supplement: Supplemental Information 1 — Adherence to and invasion of bEnd.3 cells by APEC strain (at MOI of 100). Data are means + standard errors of three independent experiments, each performed in triplicate. An asterisk indicates that the adherence and invasion values of others’ time points were significantly higher than the values of the first one hour at P-value < 0.01. [file peerj-08-9172-s001.png]
