# Supplementary material for: Transcriptome profiling of avian pathogenic Escherichia coli and the mouse microvascular endothelial cell line bEnd.3 during interaction
Source: PeerJ. 2020 May 21;8:e9172. doi: 10.7717/peerj.9172 (PMC7246031; doi:10.7717/peerj.9172)
Supplement: Supplemental Information 2 [file peerj-08-9172-s002.docx]

Table S1 bEnd.3 cell and APEC strain gene-specific primers for qRT-PCR.

| Gene name | Primers | | Log_2_ fold change  (qPCR) | Log_2_ fold change  (RNA-seq) | discription |
| --- | --- | --- | --- | --- | --- |
| bEnd.3 cell | | | | | |
| *Hilpda* | FP | GGATCACGAGGGGTCAGCTA | 1.751086 | 2.1036 | hypoxia inducible lipid droplet associated [Source:MGI Symbol;Acc:MGI:1916823] |
|  | RP | TGGAACGTGTGGTTGGACAT |  |  |  |
| *Nfkbia* | FP | CCTGACCTGGTTTCGCTCTT | 1.58104 | 2.1732 | nuclear factor of kappa light polypeptide gene enhancer in B cells inhibitor, alpha [Source:MGI Symbol;Acc:MGI:104741] |
|  | RP | AGGTAAGCTGGTAGGGGGAG |  |  |  |
| *Tnfaip3* | FP | CGACTCACCTGATCAACGCT | 1.644637 | 2.0649 | tumor necrosis factor, alpha-induced protein 3 [Source:MGI Symbol;Acc:MGI:1196377] |
|  | RP | TCGCTGTTCTCCTGCCATTT |  |  |  |
| *Apold1* | FP | GAAAGCCACCCGAAGAGTCA | 1.835812 | 2.3268 | apolipoprotein L domain containing 1 [Source:MGI Symbol;Acc:MGI:2685921] |
|  | RP | GTCAGGGACTGCATCTCACC |  |  |  |
| *Ctgf* | FP | AGACCTGTGCCTGCCATTAC | 1.217493 | 2.1209 | connective tissue growth factor [Source:MGI Symbol;Acc:MGI:95537] |
|  | RP | ACGCCATGTCTCCGTACATC |  |  |  |
| *Pxn* | FP | TCCCCAACAAGCAGAAGTCG | -0.43583 | -0.83324 | paxillin [Source:MGI Symbol;Acc:MGI:108295] |
|  | RP | CCAGTAACAGCCGGTCAAGT |  |  |  |
| *Tjp2* | FP | AGAAGAACCTCCGCAAGAGC | -0.32291 | -0.20727 | tight junction protein 2 [Source:MGI Symbol;Acc:MGI:1341872] |
|  | RP | GAACCTTCTCGTAGGCAGGG |  |  |  |
| *Lama5* | FP | TTTGGGGAACCCTTTGTGCT | -0.45766 | -1.0005 | laminin, alpha 5 [Source:MGI Symbol;Acc:MGI:105382] |
|  | RP | AGGTACAGGCCTCCGTTACT |  |  |  |
| *Ogfod1* | FP | CCATGTGCCGCATTGTTGAT | -0.55899 | -1.6362 | 2-oxoglutarate and iron-dependent oxygenase domain containing 1 [Source:MGI Symbol;Acc:MGI:2442978] |
|  | RP | GCCTCTCGGTGACTTTGAGT |  |  |  |
| *GAPDH* | FP | AACGGGAAGCCCATCACCATC | - | - | house-keeping gene for qRT-PCR |
|  | RP | AAGACACCAGTAGACTCCACGA |  |  |  |

Table S1 bEnd.3 cell and APEC strain gene-specific primers for qRT-PCR.

| Gene name | Primers | | Log2 fold change  (qPCR) | Log2 fold change  (Dual-RNA seq) | discription |
| --- | --- | --- | --- | --- | --- |
| APEC strain | | | | | |
| *clbS* | FP | GTGTAACACCAGCTCTCCCC | 0.737778 | 1.1788 | colibactin self-protection protein ClbS |
|  | RP | CCCAACTCGCCTTTGAACCT |  |  |  |
| *neuC* | FP | TACACTGGCTCCACGAACAC | 3.35 | 2.7932 | UDP-N-acetylglucosamine 2-epimerase (hydrolyzing) |
|  | RP | TGGGAACTCCTCCTCTGGTT |  |  |  |
| *tf* | FP | CACCTGCAGTCCCATCGAAA | 1.85 | 1.996 | type 1 fimbrial protein |
|  | RP | ACTGGGCCACAAGTAACGG |  |  |  |
| *rfbc* | FP | GCAGAAAGGCCAATCGATGTT | 3.177778 | 3.309 | dTDP-4-dehydrorhamnose 3,5-epimerase |
|  | RP | CCCGAAGGATTTGCTCATGG |  |  |  |
| *clbI* | FP | GATTCCCCAACTGAGCCACA | -5.20778 | -4.0402 | colibactin polyketide synthase ClbI |
|  | RP | GCAGAAGTCTCGTTAGCCCA |  |  |  |
| *clbH* | FP | CACTAACTGGCAGGTTCGGT | -3.62556 | -2.9537 | colibactin non-ribosomal peptide synthetase ClbH |
|  | RP | CCTCATGGGCTGACGATACC |  |  |  |
| *Vask* | FP | TATGGGGAATATGGGCGGGA | 0.786667 | -1.6601 | type VI secretion protein VasK |
|  | RP | TTGCGCTTTCCTTTGCTTCC |  |  |  |
| *clbC* | FP | CATCCAGGTTTTTAGCGCGG | -2.28889 | -3.0244 | colibactin polyketide synthase ClbC |
|  | RP | ATTCCACCCGCTTATTGCCA |  |  |  |
| *gapA* | FP | CGTTAAAGGCGCTAACTTCG | - | - | house-keeping gene for qRT-PCR |
|  | RP | ACGGTGGTCATCAGACCTTC |  |  |  |
