# Supplementary material for: Transcriptome profiling of avian pathogenic Escherichia coli and the mouse microvascular endothelial cell line bEnd.3 during interaction
Source: PeerJ. 2020 May 21;8:e9172. doi: 10.7717/peerj.9172 (PMC7246031; doi:10.7717/peerj.9172)
Supplement: Supplemental Information 3 [file peerj-08-9172-s003.docx]

| **Table S2 DEGs of bEnd.3 cell between the two groups** | | | | | | |
| --- | --- | --- | --- | --- | --- | --- |
|  | Components | Genes | log_2_ | | padj | Description |
|  |  |  | FoldChange | |  |  |
| **DEGs related to cell junctional complexes** | | | | | | |
| **Adherens Junctions (AJs)** | **Cadherin** | *Celsr3* | 2.124 | | 2.38E-05 | cadherin, EGF LAG seven-pass G-type receptor 3 |
|  |  | *Pcdh1* | -1.498 | | 7.29E-38 | protocadherin 1 |
|  |  | *Pcdh20* | 1.2375 | | 0.001421 | protocadherin 20 |
|  |  | *Pcdh7* | 0.71376 | | 3.13E-05 | protocadherin 7 |
|  |  | *Pcdh9* | 1.2471 | | 1.89E-07 | protocadherin 9 |
|  |  | *Pcdhb16* | 0.73662 | | 7.49E-09 | protocadherin beta 16 |
|  |  | *Pcdhb17* | 0.56758 | | 0.000489 | protocadherin beta 17 |
|  |  | *Pcdhb19* | 1.0912 | | 2.00E-05 | protocadherin beta 19 |
|  |  | *Pcdhb20* | 0.646 | | 0.002458 | protocadherin beta 20 |
|  |  | *Pcdhb21* | 1.1633 | | 2.93E-11 | protocadherin beta 21 |
|  |  | *Pcdhgc3* | -0.8528 | | 5.17E-10 | protocadherin gamma subfamily C, 3 |
| **DEGs related to actin cytoskeletal and ECMs rearrangements** | | | | | | |
| **Regulation of actin cytoskeleton** |  | *Pip5k1b* | -0.61385 | | 0.001021 | phosphatidylinositol-4-phosphate 5-kinase, type 1 beta |
|  |  | *Brk1* | -0.56007 | | 1.54E-10 | BRICK1, SCAR/WAVE actin-nucleating complex subunit |
|  |  | *Arpc1b* | -0.57732 | | 1.06E-11 | actin related protein 2/3 complex, subunit 1B |
|  |  | *Itga5* | -0.82579 | | 1.93E-23 | integrin alpha 5 (fibronectin receptor alpha) |
|  |  | *--* | -0.83023 | | 4.13E-13 | actin, cytoplasmic 1 |
|  |  | *Wasf2* | -0.85801 | | 2.73E-23 | WAS protein family, member 2 |
|  |  | *Itgb4* | -1.1033 | | 5.54E-08 | integrin beta 4 |
|  |  | *Wasl* | -1.1436 | | 5.65E-38 | Wiskott-Aldrich syndrome-like (human) |
|  |  | *Wasf1* | -1.9275 | | 1.42E-07 | WAS protein family, member 1 |
|  |  | *Pikfyve* | 0.71643 | | 4.46E-15 | phosphoinositide kinase, FYVE finger containing |
|  |  | *Vav3* | 0.65314 | | 6.16E-12 | vav 3 oncogene |
|  |  | *Diaph3* | 0.55284 | | 4.12E-08 | diaphanous related formin 3 |
| **ECM-receptor interaction** |  | *Thbs1* | 1.3407 | | 1.96E-61 | thrombospondin 1 |
|  |  | *Lamc2* | 0.81271 | | 1.22E-05 | laminin, gamma 2 |
|  |  | *Itgav* | 0.78575 | | 1.76E-21 | integrin alpha V |
|  |  | *Col5a1* | | -0.7979 | 3.47E-08 | collagen, type V, alpha 1 |
|  |  | *Itga5* | | -0.82579 | 1.93E-23 | integrin alpha 5 (fibronectin receptor alpha) |
|  |  | *Npnt* | -0.63744 | | 1.91E-07 | nephronectin |
|  |  | *Dag1* | -0.70621 | | 5.09E-16 | dystroglycan 1 |
|  |  | *Col5a3* | -0.71002 | | 0.000225 | collagen, type V, alpha 3 |
|  |  | *Col27a1* | -0.86522 | | 0.000812 | collagen, type XXVII, alpha 1 |
|  | Components | Genes | log_2_ | | padj | Description |
|  |  |  | FoldChange | |  |  |
| **ECM-receptor interaction** |  | *Lama5* | -1.0005 | | 7.90E-10 | laminin, alpha 5 |
|  |  | *Itgb4* | -1.1033 | | 5.54E-08 | integrin beta 4 |
|  |  | *Fn1* | -1.2146 | | 3.92E-30 | fibronectin 1 |
|  |  | *Agrn* | -1.25 | | 4.24E-16 | agrin |
|  |  | *Comp* | -1.5106 | | 0.042739 | cartilage oligomeric matrix protein |
|  |  | *Col1a1* | -1.7538 | | 0.001184 | collagen, type I, alpha 1 |
|  |  | *Hspg2* | -2.7127 | | 3.42E-27 | perlecan (heparan sulfate proteoglycan 2) |
|  |  | *Mmp2* | -0.65785 | | 6.61E-05 | matrix metallopeptidase 2 |
| **DEGs of immune activation and inflammatory response** | | | | | | |
| **Cytokines** |  | *Il1rap* | 0.54743 | | 2.31E-07 | interleukin 1 receptor accessory protein |
|  |  | *Il13ra1* | 0.51505 | | 0.000802 | interleukin 13 receptor, alpha 1 |
|  |  | *Il17ra* | -0.70382 | | 9.74E-11 | interleukin 17 receptor A |
|  |  | *Il3ra* | -0.86336 | | 1.61E-05 | interleukin 3 receptor, alpha chain |
|  |  | *Il6* | 1.6302 | | 9.56E-09 | interleukin 6 |
|  |  | *Il6ra* | -0.92621 | | 5.50E-22 | interleukin 6 receptor, alpha |
|  |  | *Irak1* | -0.33385 | | 0.000699 | interleukin-1 receptor-associated kinase 1 |
|  |  | *Irak2* | 0.55172 | | 1.72E-07 | interleukin-1 receptor-associated kinase 2 |
|  |  | *Nfkbia* | 2.1732 | | 1.68E-25 | nuclear factor of kappa light polypeptide gene enhancer in B cells inhibitor, alpha |
|  |  | *Nod2* | 0.67657 | | 0.010291 | nucleotide-binding oligomerization domain containing 2 |
|  |  | *Tnfaip3* | 2.0649 | | 0.000554 | tumor necrosis factor, alpha-induced protein 3 |
|  |  | *Prkcg* | -1.8984 | | 1.98E-39 | protein kinase C, gamma |
|  |  | *Mapk11* | -0.55838 | | 7.40E-05 | mitogen-activated protein kinase 11] |
|  |  | *Mapk12* | -0.72561 | | 3.26E-12 | mitogen-activated protein kinase 12 |
|  |  | *Tab2* | -0.74311 | | 1.20E-17 | TGF-beta activated kinase 1/MAP3K7 binding protein 2 |
|  |  | *Tab1* | -0.93564 | | 1.16E-14 | TGF-beta activated kinase 1/MAP3K7 binding protein 1 |
|  |  | *Mapk3* | -1.0974 | | 1.33E-21 | mitogen-activated protein kinase 3 |
|  |  | *Rela* | -1.1492 | | 3.11E-10 | v-rel reticuloendotheliosis viral oncogene homolog A (avian) |
|  |  | *Card6* | -2.2609 | | 5.53E-21 | caspase recruitment domain family, member 6 |
|  |  | *Ncf2* | 3.0239 | | 0.00011772 | neutrophil cytosolic factor 2 |
|  |  | *Vcam1* | 0.63813 | | 0.006226 | vascular cell adhesion molecule 1 |
