# Supplementary material for: Transcriptome profiling of avian pathogenic Escherichia coli and the mouse microvascular endothelial cell line bEnd.3 during interaction
Source: PeerJ. 2020 May 21;8:e9172. doi: 10.7717/peerj.9172 (PMC7246031; doi:10.7717/peerj.9172)
Supplement: Supplemental Information 4 [file peerj-08-9172-s004.docx]

| **Table 3 DEGs of APEC strain between two groups** | | | | | |
| --- | --- | --- | --- | --- | --- |
|  | Components | Gene | log_2_ | padj | Description |
|  |  | name | FoldChange |  |  |
| **Virulence Factors related to meningitis** | | | | | |
| inner membrane protein | CXG97_RS21095 | *yhjD* | -1.2409 | 0.02386 | inner membrane protein YhjD |
|  | CXG97_RS04730 | *-* | -3.7635 | 5.98E-12 | inner membrane transport permease YbhR |
|  | CXG97_RS20845 | *-* | -2.7222 | 3.62E-06 | inner membrane transport permease YhhJ |
| colicin | CXG97_RS26810 | *-* | 1.5822 | 0.0023363 | colicin |
|  | CXG97_RS27750 | *-* | 2.3002 | 2.80E-05 | colicin B immunity protein |
|  | CXG97_RS27740 | *-* | 2.0799 | 7.62E-05 | colicin M |
|  | CXG97_RS26820 | *-* | 1.406 | 0.0088784 | colicin V secretion protein CvaA |
|  | CXG97_RS26815 | *-* | 2.8622 | 0.00013252 | colicin V secretion protein CvaB |
| colibactin | CXG97_RS11495 | *clbS* | 1.1788 | 0.034169 | colibactin self-protection protein ClbS |
|  | CXG97_RS11550 | *clbG* | -2.845 | 1.09E-07 | colibactin biosynthesis acyltransferase ClbG |
|  | CXG97_RS11525 | *clbL* | -1.6486 | 0.0025406 | colibactin biosynthesis amidase ClbL |
|  | CXG97_RS11555 | *clbF* | -2.5715 | 1.24E-06 | colibactin biosynthesis dehydrogenase ClbF |
|  | CXG97_RS11500 | *clbQ* | -1.0918 | 0.046382 | colibactin biosynthesis thioesterase ClbQ |
|  | CXG97_RS11575 | *clbB* | -1.6643 | 0.0011995 | colibactin hybrid non-ribosomal peptide synthetase/type I polyketide synthase ClbB |
|  | CXG97_RS11530 | *clbK* | -1.1324 | 0.033288 | colibactin hybrid non-ribosomal peptide synthetase/type I polyketide synthase ClbK |
|  | CXG97_RS11545 | *clbH* | -2.9537 | 1.47E-08 | colibactin non-ribosomal peptide synthetase ClbH |
|  | CXG97_RS11535 | *clbJ* | -2.896 | 2.46E-08 | colibactin non-ribosomal peptide synthetase ClbJ |
|  | CXG97_RS11515 | *clbN* | -1.6232 | 0.0018144 | colibactin non-ribosomal peptide synthetase ClbN |
|  | CXG97_RS11570 | *clbC* | -3.0244 | 7.15E-09 | colibactin polyketide synthase ClbC |
|  | CXG97_RS11540 | *clbI* | -4.0402 | 2.55E-14 | colibactin polyketide synthase ClbI |
|  | CXG97_RS11510 | *clbO* | -1.6668 | 0.0019112 | colibactin polyketide synthase ClbO |
|  |  |  |  |  |  |
|  |  |  |  |  |  |

|  | Components | Gene | log_2_ | padj | Description |
| --- | --- | --- | --- | --- | --- |
|  |  | name | FoldChange |  |  |
| type VI secretion protein | CXG97_RS16800 | *-* | -1.6601 | 0.015351 | type VI secretion protein VasK |
|  | CXG97_RS01210 | *clpV* | -2.6525 | 2.93E-05 | type VI secretion system ATPase TssH |
|  | CXG97_RS01195 | *icmF* | -1.9201 | 0.0020067 | type VI secretion system membrane subunit TssM |
|  | CXG97_RS01185 | *-* | -1.5031 | 0.033181 | type VI secretion system tube protein Hcp |
| ABC transporters | CXG97_RS01085 | *metN* | -4.95 | 6.60E-05 | methionine import ATP-binding protein MetN |
|  | CXG97_RS28455 | *-* | -4.0682 | 0.000347 | ABC transporter substrate-binding protein |
|  | CXG97_RS28445 | *-* | -3.5279 | 0.000718 | ABC transporter ATP-binding protein |
|  | CXG97_RS04820 | *-* | -3.0871 | 6.09E-09 | glutamine ABC transporter permease |
|  | CXG97_RS10720 | *-* | -2.8896 | 0.049694 | zinc ABC transporter substrate-binding protein |
|  | CXG97_RS28450 | *-* | -2.8313 | 0.002204 | iron ABC transporter permease |
|  | CXG97_RS05280 | *potG* | -2.7288 | 1.35E-05 | polyamine ABC transporter ATP-binding protein |
|  | CXG97_RS00375 | *thiB* | -2.6589 | 0.019117 | thiamine ABC transporter substrate binding subunit |
|  | CXG97_RS11025 | *-* | -2.4912 | 2.07E-05 | amino acid ABC transporter permease |
|  | CXG97_RS09950 | *-* | -2.4497 | 3.09E-05 | vitamin B12 import ATP-binding protein BtuD |
|  | CXG97_RS19285 | *lptB* | -2.3178 | 2.21E-05 | ABC transporter ATP-binding protein |
|  | CXG97_RS12610 | *-* | -2.2572 | 0.001108 | ABC transporter permease |
|  | CXG97_RS08195 | *sapF* | -2.242 | 0.00014 | peptide ABC transporter ATP-binding protein SapF |
|  | CXG97_RS20615 | *ugpC* | -2.1846 | 0.000463 | sn-glycerol-3-phosphate import ATP-binding protein UgpC |
|  | CXG97_RS24075 | *-* | -2.167 | 0.007543 | maltose ABC transporter permease |
|  | CXG97_RS04815 | *glnQ* | -1.8469 | 0.000443 | glutamine ABC transporter ATP-binding protein |
|  | CXG97_RS12605 | *-* | -1.7836 | 0.005834 | ABC transporter ATP-binding protein |

|  | Components | Gene | log_2_ | padj | Description |
| --- | --- | --- | --- | --- | --- |
|  |  | name | FoldChange |  |  |
| ABC transporters | CXG97_RS07595 | *-* | -1.774 | 0.001011 | oligopeptide ABC transporter ATP-binding protein OppF |
|  | CXG97_RS04115 | *modB* | -1.752 | 0.005234 | molybdate ABC transporter permease subunit |
|  | CXG97_RS04825 | *-* | -1.7477 | 0.000984 | amino acid ABC transporter substrate-binding protein |
|  | CXG97_RS13005 | *ccmB* | -1.7151 | 0.030047 | heme exporter protein CcmB |
|  | CXG97_RS07590 | *oppD* | -1.7141 | 0.002087 | oligopeptide ABC transporter ATP-binding protein OppD |
|  | CXG97_RS05475 | *-* | -1.7055 | 0.001961 | cysteine/glutathione ABC transporter ATP-binding protein/permease CydC |
|  | CXG97_RS19660 | *-* | -1.6942 | 0.003137 | amino acid ABC transporter permease |
|  | CXG97_RS04930 | *-* | -1.5863 | 0.003158 | glutathione-binding protein GsiB |
|  | CXG97_RS05275 | *-* | -1.4824 | 0.005951 | spermidine/putrescine ABC transporter substrate-binding protein PotF |
|  | CXG97_RS21005 | *-* | -1.4784 | 0.008161 | iron ABC transporter permease |
|  | CXG97_RS19250 | *-* | -1.4667 | 0.00647 | phospholipid ABC transporter permease |
|  | CXG97_RS08205 | *sapC* | -1.4476 | 0.029618 | peptide ABC transporter permease SapC |
|  | CXG97_RS08215 | *-* | -1.4156 | 0.01374 | peptide ABC transporter substrate-binding protein SapA |
|  | CXG97_RS16020 | *-* | -1.3503 | 0.024248 | proline/glycine betaine ABC transporter permease ProW |
|  | CXG97_RS12900 | *-* | -1.3272 | 0.025785 | microcin C ABC transporter ATP-binding protein YejF |
|  | CXG97_RS01080 | *-* | -1.3062 | 0.016791 | methionine ABC transporter |
|  | CXG97_RS21255 | *-* | -1.2671 | 0.016235 | periplasmic dipeptide transporter |
|  | CXG97_RS03270 | *-* | -1.246 | 0.035868 | iron-enterobactin transporter permease |
|  | CXG97_RS10925 | *araG* | -1.2236 | 0.024914 | arabinose import ATP-binding protein AraG |
|  | CXG97_RS04110 | *-* | -1.181 | 0.036125 | molybdate ABC transporter substrate-binding protein |

|  | Components | Gene | log_2_ | padj | Description |
| --- | --- | --- | --- | --- | --- |
|  |  | name | FoldChange |  |  |
| ABC transporters | CXG97_RS13540 | *-* | 1.3318 | 0.017068 | histidine ABC transporter permease |
|  | CXG97_RS08965 | *-* | 1.3351 | 0.027774 | ABC transporter ATP-binding protein/permease |
|  | CXG97_RS11035 | *-* | 1.3622 | 0.009897 | cystine-binding periplasmic protein |
|  | CXG97_RS17970 | *-* | 1.4492 | 0.005925 | polysialic acid transporter |
|  | CXG97_RS17965 | *-* | 1.7481 | 0.01082 | polysialic acid transport ATP-binding protein KpsT |
|  | CXG97_RS13550 | *-* | 1.9427 | 0.000172 | histidine ABC transporter substrate-binding protein HisJ |
|  | CXG97_RS18120 | *lptG* | 2.0969 | 0.000743 | LPS export ABC transporter permease LptG |
|  | CXG97_RS13545 | *-* | 2.1844 | 2.66E-05 | histidine ABC transporter permease |
|  | CXG97_RS23390 | *-* | 2.5348 | 1.41E-05 | sulfate ABC transporter substrate-binding protein |
|  | CXG97_RS05305 | *-* | 7.4588 | 5.85E-36 | arginine/ornithine ABC transporter substrate-binding protein |
| Phosphotransferase system (PTS) | CXG97_RS10080 | *-* | 1.6662 | 0.027313 | N%2CN'-diacetylchitobiose-specific phosphotransferase enzyme IIA component |
|  | CXG97_RS10085 | *-* | 2.1031 | 0.005364 | PTS N%2CN'-diacetylchitobiose transporter subunit IIC |
|  | CXG97_RS10090 | *-* | 2.4253 | 6.73E-05 | PTS sugar transporter subunit IIB |
|  | CXG97_RS10510 | *-* | -1.3423 | 0.011381 | PTS mannose/fructose/sorbose transporter subunit IIC |
|  | CXG97_RS10515 | *-* | -1.1522 | 0.032667 | PTS mannose transporter subunit IID |
|  | CXG97_RS12825 | *-* | -1.7716 | 0.000638 | PTS fructose transporter subunit IIBC |
|  | CXG97_RS12835 | *-* | -2.7947 | 1.08E-06 | multiphosphoryl transfer protein |
|  | CXG97_RS16210 | *-* | -1.4079 | 0.031494 | PTS cellobiose/arbutin/salicin transporter subunit IIBC |
|  | CXG97_RS16940 | - | -1.9443 | 0.00023 | phosphoenolpyruvate-protein phosphotransferase PtsP |
|  | CXG97_RS26010 | *ptsP* | -1.2791 | 0.026796 | phosphoenolpyruvate--protein phosphotransferase |

|  | Components | Gene | log_2_ | padj | Description |
| --- | --- | --- | --- | --- | --- |
|  |  | name | FoldChange |  |  |
| **Proteins export and amino acid metabolism** | | | | | |
| Histidine metabolism | CXG97_RS12020 | *-* | -2.5773 | 6.60E-07 | bifunctional histidinol-phosphatase/imidazoleglycerol-phosphate dehydratase HisB |
|  | CXG97_RS12025 | *hisH* | -2.6139 | 1.39E-06 | imidazole glycerol phosphate synthase subunit HisH |
|  | CXG97_RS12010 | *hisD* | -1.966 | 0.00012352 | histidinol dehydrogenase |
|  | CXG97_RS12030 | *hisA* | -2.0592 | 0.00014003 | 1-(5-phosphoribosyl)-5-((5-phosphoribosylamino)methylideneamino)imidazole-4-carboxamide isomerase |
|  | CXG97_RS12015 | *-* | -2.5077 | 0.00014602 | histidinol-phosphate transaminase |
|  | CXG97_RS12035 | *hisF* | -1.521 | 0.0043749 | imidazole glycerol phosphate synthase subunit HisF |
| Valine, leucine and isoleucine biosynthesis | CXG97_RS00395 | *leuC* | -2.8174 | 6.03E-08 | 3-isopropylmalate dehydratase large subunit |
|  | CXG97_RS00400 | *leuB* | -1.7713 | 0.00076136 | 3-isopropylmalate dehydrogenase |
|  | CXG97_RS22545 | *-* | -1.7238 | 0.00078849 | dihydroxy-acid dehydratase |
|  | CXG97_RS22540 | *-* | -1.6477 | 0.0017483 | branched-chain-amino-acid transaminase |
|  | CXG97_RS22550 | *ilvA* | -1.602 | 0.0027344 | threonine ammonia-lyase%2C biosynthetic |
|  | CXG97_RS22535 | *-* | -2.2384 | 0.011223 | acetolactate synthase isozyme 2 small subunit |
|  | CXG97_RS22530 | - | 1.1015 | 0.04277 | acetolactate synthase 2 catalytic subunit |
|  | CXG97_RS00420 | - | -1.0986 | 0.048221 | acetolactate synthase 3 large subunit |
